# Cultural safety, the LGBTQI+ community and international medical graduate training

**DOI:** 10.5694/mja2.52617

**Published:** 2025-03-16

**Authors:** Cindy Towns, Charlene Rapsey, Rhea Liang

**Affiliations:** ^1^ University of Otago, Wellington Wellington New Zealand; ^2^ Capital and Coast District Health Board Wellington New Zealand; ^3^ University of Otago Dunedin New Zealand; ^4^ Robina Hospital Gold Coast QLD; ^5^ Bond University Gold Coast QLD

**Keywords:** Cultural competency, Healthcare disparities, Gender identity, Homosexuality, Education, medical, continuing, Global health, Social Determinants of Health, Sexual health, Medical education

Culturally safe health care for all people is a requirement for medical practice in Australia and Aotearoa New Zealand.^1^, ^2^ In both countries, legislation protects the rights of the lesbian, gay, bisexual, transgender, queer and intersex (LGBTQI+) community. Despite progress toward equality, higher rates of discrimination towards LGBTQI+ communities contribute to double the risk of mental health disorders and increased inequities in health outcomes, such as cardiovascular disease and cancer survivorship, compared with their non‐LGBTQI+ counterparts.^3^, ^4^, ^5^

It is unacceptable that LGBTQI+ patients continue to report discriminatory and inadequate medical care.^6^, ^7^ Discrimination within the health care system leads to avoidance of care, amplifying negative health consequences.^8^ For example, trans patients who experience discrimination are more likely to avoid preventive and urgent health care services than trans patients who do not experience discrimination.^9^ To address these inequities, it is essential that medical training includes specific education on LGBTQI+ health care needs.

Many of the international medical graduates (IMGs) entering Australasia to practise come from countries that reject and criminalise LGBTQI+ communities. Therefore, education and clinical exposure for IMGs who have not received undergraduate training or exposure to LGBTQI+ communities are a priority. The lack of experience, knowledge and understanding compromises patient care and risks worsening health inequities.^10^ Compromised care also exposes IMGs to the risk of complaint and sanction. Given the reliance of both countries on IMGs, there is an urgent need for additional training and assessment for these doctors.

## Registration requirements

Medical Councils in Australasia require respectful and responsive care for LGBTQI+ communities. Specifically, the Medical Board of Australia “requires genuine efforts to adapt your practice as needed, to respect diversity and avoid bias, discrimination and racism. It also involves challenging assumptions that may be based on, for example, gender, disability, race, ethnicity, religion, sexuality, age or political beliefs”.^2^ The Australian Medical Council recognises the lesbian, gay, bisexual, trans, queer, asexual, intersex and questioning (LGBTQAI+) community as a group experiencing specific health inequity and expects partnership with stakeholders and local community.^11^ Their expectations are that medical practitioners “… are aware of their own culture and beliefs and respectful of the beliefs and cultures of others, recognising that these cultural differences may impact on the doctor–patient relationship and on the delivery of health services”.

In Aotearoa New Zealand, cultural competency for medical practitioners is legally required.^1^ Further, the Medical Council of New Zealand (MCNZ) specifies gender and sexual identities within the definition of culture^12^ and has previously defined cultural safety as ensuring that “a doctor has the attitudes, skills and knowledge needed to function effectively and respectfully when … treating people of different cultural backgrounds”.^13^ The MCNZ now uses the broader term cultural safety and defines that as:

The need for doctors to examine themselves and the potential impact of their own culture on clinical interactions and healthcare service delivery. The commitment by individual doctors to acknowledge and address any of their own biases, attitudes, assumptions, stereotypes, prejudices, structures and characteristics that may affect the quality of care provided.^14^

## Legislative context

In Aotearoa New Zealand, the Homosexual Law Reform Bill (1986) decriminalised sex between consenting male adults. The *Marriage Equality Act 2013* gave same sex couples legislative equality. In 2017, the Criminal Records Bills expunged historical criminal offences for same sex activity and, from 2018, same sex parents were recognised on birth certificates. The *Human Rights Act 1993* prohibits discrimination on the basis of sex. Self‐identification of gender is accepted for passports and birth certificates. The *Conversion Practices Prohibition Legislation Act 2022* outlawed conversion treatments for sexual and gender minorities.

In Australia, federal decriminalisation of homosexual activity occurred in 1994,^15^ federal marriage equality was achieved in 2017,^16^ and discrimination on the basis of sex, including non‐binary sex, is protected under the *Sex Discrimination Act 1984*. Conversion therapy (also known as reparative therapy), which is a range of harmful practices that falsely claim to change a person's sexual orientation or gender identity or expression, is banned in four states (Victoria, Queensland, the Australian Capital Territory and New South Wales), and a ban is being considered in Tasmania.

In stark comparison to Australasia are the 61 countries that continue to criminalise same sex conduct (eg, Algeria, Guyana, Bangladesh, Turkmenistan, Afghanistan, Brunei).^17^ Of these, at least seven countries retain the death penalty, including Brunei, Iran, Mauritania, Saudi Arabia, Nigeria, Uganda and Yemen.^17^ Although capital punishment may be limited to men and specific sexual acts, it remains deeply concerning. Several countries also criminalise forms of gender expression including Brunei, Malawi, Malaysia, Oman, Saudi Arabia, South Sudan, Tonga and the United Arab Emirates. Some countries that do not have a federal law still criminalise sexual and gender minorities under Sharia law (eg, Malaysia and Nigeria). Saudi Arabia has no codified law, but police will arrest people based on their gender expression.^18^

## International medical graduate workforce

Medical workforce surveys document Australasia's heavy reliance on IMGs.^19^ In Australia, IMGs comprised 28.8% of the workforce, with a higher representation in rural and regional areas and in general practice than in other locations and specialties.^20^ Given the comparative lack of queer resources in rural communities, it is arguably even more important for IMGs in these areas to be able to provide culturally safe practice. In Aotearoa New Zealand, over 40% of registered doctors were international graduates;^21^ with the highest proportions in primary practice (50%), obstetrics and gynaecology (50%), and psychiatry (60%). In Aotearoa New Zealand, IMGs come from over 100 different countries, with those most likely to remain permanently coming from areas most likely to discriminate against the LGBTQI+ community.^18^ Given increasing referrals for gender‐affirming care,^22^ and the complexity of decision making around gender transition in adolescence, it is vital that doctors have appropriate experience, education, and assessment skills.

## Educational context

Although criticised for not providing enough material on gender minorities, all New Zealand medical students and most Australian medical students receive formal teaching on LGBTQI+ health care.^23^, ^24^ Curriculum content includes teaching on the role of stigma, discrimination and violence in poor health outcomes as well as introducing skills important for clinical practice, such as respectful pronoun use.^23^, ^24^ In addition, clinical placements include care for LGBTQI+ people in hospital and community settings. The value of this teaching is supported by evidence that suggests it can change knowledge and attitudes^25^ and calls by medical students for specific content to be mandated across all undergraduate programs.^26^

Further, for IMGs, despite the legal requirement for cultural competency and MCNZ guidelines on cultural safety, there is no specific teaching on LGBTQI+ cultural safety in postgraduate years (PGY) 1 and 2 for doctors — the first author (CT) taught the only PGY1 course in New Zealand on this subject, but this was withdrawn in 2023 due to concerns that it was insufficient for IMGs who come from countries that criminalise gender and sexual minorities. In Aotearoa New Zealand, IMGs entering pre‐vocational practice must come through the New Zealand registration exam, which does not include a specific LGBTQI+ cultural safety training or assessment. LGBTQI+ cultural safety therefore relies on what the doctors were exposed to in their undergraduate education and the cultural and legislative context in which they grew up.

IMGs coming from countries that criminalise gender and sexual minorities have likely had no opportunity for training in health care for LGBTQI+ communities. Further, regardless of whether IMGs agree with the laws or cultural norms in their country of origin, they will probably not have experience dealing with the health care needs of this population in places where it is illegal to identify as a member of the LGBTQI+ community. This lack of experience will affect understanding of queer language (Supporting Information), health needs, queer relationships, sexual behaviours, fertility, and family planning, and has the potential to exacerbate well documented health disparities. Lack of knowledge and skills puts clinicians at risk of causing harm to patients and for consequent avoidance of the health system by LGBQTI+ communities. Correspondingly, even unintentional discriminatory practice puts IMGs at risk for complaint, which can take a significant toll on their wellbeing and career.^27^

Although this perspective article focuses on IMGs and the legislative and educational differences between Australasia and other countries, we recognise that locally trained doctors will not always provide culturally safe practice or refrain from perpetuating discrimination. Given a lack of specific cultural safety in continuing medical education (especially for late career doctors) and the variability in education across Australasian curricula, there is a strong argument for mandated undergraduate and postgraduate training for all doctors practising in Australia and Aotearoa New Zealand.

## Recommendations

LGBTQI+ health care training and assessment should be mandated for IMGs entering practice in Australasia. In countries where sexual and gender identity expression are criminalised, there is limited opportunity for doctors to gain understanding and experience of LGBTQI+ patients and their health needs. For medical governance bodies to reasonably expect professional guidelines to be followed, education is essential. Given the current workforce crisis and heavy reliance on IMGs, this training shortfall should be addressed with urgency.

## Competing interests

No relevant disclosures.

## Provenance

Not commissioned; externally peer reviewed.

## Supporting information


Supplementary glossary
