# Supplementary material for: Cultural safety, the LGBTQI+ community and international medical graduate training
Source: Med J Aust. 2025 Mar 16;222(8):384–6. doi: 10.5694/mja2.52617 (PMC12050245; doi:10.5694/mja2.52617)
Supplement: Supplementary file 1 — Supplementary glossary [file MJA2-222-384-s001.pdf]

MJA
The Medical Journal of Australia

Supporting Information

Supplementary material

This appendix was part of the submitted manuscript and has been peer reviewed.
It is posted as supplied by the authors.

Appendix to: Towns C, Rapsey C, Liang R. Cultural safety, the LGBTQI+ community and international medical graduate training. *Med J Aust* 2025; doi: 10.5694/mja2.52617.

Glossary of queer terms

Term	Definition
Asexual	A spectrum in which someone experiences little to no sexual attraction/desire for others.
Bisexual	Commonly refers to attraction to men and women but more inclusively defines someone who is attracted to more than one gender.
Cisgender (cis)	A term for people whose gender identity aligns with the sex assigned to them at birth.
Gay	A person who is emotionally, romantically, and/or sexually attracted to people of the same gender.
Gender diverse/diversity	An umbrella term referring to gender identity or gender expression which does not conform to cisgender norms, (includes trans, non-binary and genders from other cultures).
Gender dysphoria	The incongruence, dissonance, disconnect, or discomfort with one's expressed or experienced gender and the assigned at birth and/or one's body/sex characteristics.
Gender expression/presentation	The physical or outward expression of one's gender through clothing, hairstyle, voice, makeup, body shape, mannerisms etc.
Gender/gender identity	One's sense of self relating to gender, based on our social roles, expectations, and assumptions, such as being male/man, female/woman, neither, or another identity.
Gender-affirming care	Healthcare that affirms and validates a person's gender. May include mental health care, support with social transition, hormonal or surgical treatments.
Heterosexual/straight	A person who exclusively experiences emotional, romantic, and/or sexual attraction to those of the opposite gender.
Homophobia	Broad term referring to discrimination, intolerance, discomfort, or stereotyping of non-heterosexual people.
Homosexual	A person who experiences emotional, romantic, and/or sexual attraction to those of the same gender.
Intersex	A broad umbrella term describing sex characteristic variations such as chromosomes, hormones, genitals, and/or secondary sex characteristics.
Lesbian	Women who are emotionally, romantically, and/or sexually attracted to women.
LGBT, LGBTQ, LGBTQIA+ and variations	An acronym referring to lesbian, gay, bisexual, transgender, queer, intersex, asexual/aromantic people. Used as an umbrella term to refer to all people with diverse sexualities and genders.

Non-binary	A term describing people whose gender falls outside the binary categories of male/man/boy and female/woman/girl.
Pansexual	Attraction to people of any or all genders, regardless of their gender, gender expression, or sex characteristics.
Pronouns	Words used to refer to people in the third person as a substitute for their name such as she/her, he/him, and they/them.
Queer	A reclaimed umbrella term referring to diverse sexualities and genders; gender and sexual minorities.
Questioning	Someone who is questioning and/or exploring their gender identity or sexuality.
Rainbow	An umbrella term referring to people of diverse sexualities, genders, and sex characteristics.
Sexual orientation/sexuality	A person's sexual identity, behaviour, and/or attractions in relation to the people they are attracted to.
Sex characteristics	Primary sex characteristics include chromosomes, sex hormones, and reproductive organs. Secondary sex characteristics include breast hair and voice changes.
Sex	The biological and physiological characteristics that define humans as female or male. Usually assigned at birth. Not mutually exclusive – characteristics can coexist (see intersex).
The gender binary	The perception that gender consists of two categories, male/man and female/woman, in which no other possibilities for gender exist.
Transgender (trans)	A term describing a person whose gender differs from the sex or gender that was assigned to them at birth.
